# Pediatric self-medication use in Rwanda – a cross sectional study

**DOI:** 10.4314/ahs.v20i4.61

**Published:** 2020-12

**Authors:** Joyeuse Ukwishaka, Christian Umuhoza, Peter Cartledge, Natalie McCall

**Affiliations:** 1 University of Rwanda; 2 University Teaching Hospital of Kigali; 3 Yale University (USA), Rwanda Human Resources for Health (HRH) Program, Rwanda

**Keywords:** Self-medication, medicines, parents, caregivers, children, Nonprescription Drugs, Rwanda

## Abstract

**Background:**

Self-medication, a worldwide practice, has both benefits and risks. Many countries have regulated non-prescription medications available for use in self-medication. However, in countries such as Rwanda, where prescriptions are not required to purchase medications, prescription, non-prescription and traditional medications have been used for self-medication.

**Objectives:**

To determine the reported self-medication use in Rwanda and to determine attitudes and reasons associated with parental decisions to self-medicate their children.

**Methods:**

A cross-sectional multi-center questionnaire based quantitative study of 154 parents/caregivers of children under ten years undertaken in private and public health facilities.

**Results:**

The use of self-medication was reported to be 77.9%. Among these parents/caregivers, 50.8% used modern self-medication only, 15.8% used traditional self-medication only and 33.3% used both types of self-medication. Paracetamol was the most commonly used drug in modern self-medication; the traditional drugs used were Rwandan local herbs. Parents/caregivers who used modern medicines had slightly more confidence in self-medication than self-medication users of traditional medicines (p=0.005). Parents/caregivers who used modern self-medication reported barriers to consultation as a reason to self-medicate more frequently than those who used traditional drugs. Having more than one child below 10 years of-age was the only socio-demographic factor associated with having used self-medication (AOR=4.74, CI: 1.94–11.58, p=0.001). Being above 30 years (AOR= 5.78, CI: 1.25–26.68, p=0.025) and living in Kigali (AOR=8.2, CI: 1.58–43.12, p=.0.012) were factors associated with preference of modern self- medication compared to traditional self-medication.

**Conclusion:**

Self-medication is common in Rwanda. Parents/caregivers are involved in this practice regardless of their socio-demographic background.

## Introduction

Since the declaration of Alma-Ata in 1978, the principles of self-care, individual participation, responsibility and involvement in one's own health care have been recognized as important elements of primary healthcare^1^–^3^. Self-care includes self-medication which the WHO defines as “the selection and use of medicines by individuals to treat self-recognized illnesses or symptoms”^4^–^7^. In the pediatric population, self-medication implies administration of a drug to a child, by a caregiver, without prior medical consultation 8. WHO defines traditional medicine as “The sum of knowledge, skill, and practices based on the theories, beliefs, and experiences indigenous to different cultures, whether explicable or not, used in the maintenance of health as well as in the prevention, diagnosis, improvement or treatment of physical and mental illness” 9,10. Traditional medicines will often be chosen and provided by a traditional healer or therapist, but can also be produced or purchased by an individual and used as a form of self-medication.

Responsible self-medication implies the use of approved, safe, and effective drugs, accompanied by information to direct users 11. There are several advantages of responsible self-medication, but the population must be aware of potentials risks and harms of self-medication^12^,^13^.

Factors associated with the parental choice to self-medicate their children vary, but include: parents/caregivers perceiving their child's illness as being mild and not requiring health professional consultation, lack of time to attend consultations, high consultation fees, clinic waiting time, emergency treatment, use of old prescriptions available in the home, parent comfort in recognizing their children's disease based on the symptoms and having experience with the medication 14,15.

Pediatric self-medication is a worldwide practice with a reported prevalence between 32–98% in Madagascar, India, Greece, and Australia 8,16–18. Tanzania, a fellow nation of the East African community has reported pediatric self-medication rates of 69% 19.

There is also a difference in education and economic factors associated with the choice to self-medicate. Studies from Spain, Finland, Italy, and Madagascar^12^,^19^–^21^, found that families with high income and with secondary and higher education level practice more self-medication than families with low income and low education level. On the other hand, in studies done in Germany and India, family income and parental education were not significantly influencing self-medication practice 9,22.

There is insufficient literature describing the use of self-medication in pediatric populations in Sub-Saharan Africa. In Rwanda, self-medication is being used by parents/caregivers for their children 20,21. The Rwandan Ministry of Health regulates essential medicines in accordane with WHO recommendations but there is no regulation of non-prescription or OTC drugs. In addition, there is a rise in antibiotic resistance in the country. Self-medication, if not done in a responsible and safe manner, could predispose the population to more harm than benefits. So there is a need to establish a baseline data on parental self-medication practices in Rwanda including the use of traditional and modern drugs. This could help health care providers and policymakers to plan education and establish policies aiming to achieve responsible use of medicines.

**Objectives** The present study aimed at determining the proportion of parents/caregivers who reported self-medicating their children before consulting private and public health facilities in Rwanda and to determine attitudes and reasons associated with parental decisions to self-medicate their children.

## Methods

**Study Design:** We conducted a cross-sectional study from July to September 2018. Reporting has been verified in accordance with the STROBE (Strengthening the Reporting of Observational Studies in Epidemiology) checklist 22.

**Study Sites/settings:** We conducted a multi-center study in three facilities: i) a provincial referral hospital, two hours from Kigali, the capital of Rwanda, ii) a vaccination clinic in an urban public health center in Kigali city and iii) a private pediatric clinic in Kigali, which serves a larger proportion of educated parents/caregivers with high socioeconomic level. This selection was done to increase the representativeness and generalizability of the results to the Rwandan Population.

**Study population:** The study aimed to recruit parents/caregivers of both unwell and well children with different socioeconomic status. Unwell children were recruited at out and in-patient units of one private and one public health facility. Parents/caregivers of well children were approached at a vaccination clinic in a health center.

**Inclusion criteria:** All Rwandan parents/caregivers of children aged one month to 10 years of life and who accepted to participate in the study were eligible to be included. Parents/caregivers who were not primary caregivers of the children were excluded as they would not be able to describe fully the self-treatment of the children. We excluded from the analysis the participants who consented but did not fully complete the questionnaire.

**Sampling:** Non-probability convenience sampling was used to recruit study subjects on working days (Monday to Friday) during working hours (8am to 5pm).

**Procedures for enrollment:** Eligible parents/caregivers were approached in the waiting area, prior to their consultation, and they were given a verbal explanation of the purpose and the methods of the study. All participants who signed the written consent form were then included. No data was kept on the number of participants who declined to participate.

**Administering the questionnaire:** Questionnaires were given to parents/caregivers by the PI or a trained data collector (DC) who was available to answer questions regarding the questionnaire. For parents/caregivers who couldn't read or who preferred to be assisted, the DC read the questions and completed the questionnaires on behalf of the participant. The questionnaires were completed in the waiting area of the respective health facility. When the questionnaire was filled by the DC, this was indicated on the form.

**Variables:** Dependent (outcome) variable were the percentage of parents/caregivers using self-medication, reasons of parental self-medication and common drugs used in self-medication. Independent variables were age, sex, province of origin, level of education, socioeconomic status, number of children under 10 years of age; health insurance, determinant/predictors of parental self-medication, relationship with the child, marital status. Potential confounders were site of interview and mode of completing questionnaire (verbal or written)

**Data collection tool (Questionnaire):** A questionnaire was developed specifically for this study. To ensure content validity of our data collection tool, it was first built by the Principal Investigator (PI) (JU) using previously published studies^14^,^15^,^23^–^25^. The first draft was then reviewed by four local experts in research (three pediatricians (CU, NM & PC) and one statistician). There were four sections to the questionnaire, namely: demo- graphic characteristics of respondents; modern medicine questionnaire; traditional medicine questionnaire and finally the no self-medicating questionnaire. Parents/caregivers were asked whether they have ever used self-medication for their children and if so, what type they had used. Depending on their answers, they completed the appropriate questionnaire (i.e. Modern and/or Traditional medicines) or the questionnaire for parents/caregivers who have never used self-medication for their children. Therefore, not all subjects completed all sections of the questionnaires

The questionnaires used 5-point Likert-scales to assess the parental attitudes and perceptions about self-medication. The responses given to the Likert-scale questions from each of the barriers (five questions) and confidence (six questions) domains were combined to create total “barriers to consultation” and total “confidence in self-medicating” scores.

**Translation and piloting of questionnaires:** The questionnaire was initially prepared in English and translated into the national language (Kinyarwanda) by the PI. It was then back-translated in English for accuracy by an independent, non-medical translator. It was piloted on five subjects and amendments were then made based on this piloting, including changes in wording and the addition of further Likert-items.

**Sample size calculation:** Sample-size was calculated for the prevalence of self-medicating, with a predicted baseline of 69% of parents/caregivers of children under-five years of age reported in Tanzania 19. Using the formula with Finite Population Correction 26 and a level of confidence aimed at 95% we calculated the sample size to be 152 study participants.

**Data management:** EpiData Entry 3.1 Software was used for data entry and storage. The data was then exported into Statistical Package for the Social Sciences (IBM Corp. Released 2011. IBM SPSS Statistics for Windows, Version 20.0. Armonk, NY: IBM Corp.), for statistical analysis.

**Statistical analysis:** For continuous numerical data, means and standard deviation were reported; for categorical data, frequency tables, percentages and graphs were reported. Comparison was made between responses on the modern-medicine and traditional-medicine questionnaires. Therefore, parents/caregivers, who self-medicated with both modalities, were included in both arms of the analysis For confidence and barrier questions, Likert-scale items were converted into means and compared using Mann-Whitney U test due to the non-normal distribution of the data. Categorical variables were analyzed using Chi-square test or Fisher's exact test. Odds ratio were reported to identify associations. To account for multiple confounders logistic regression analysis was undertaken including variables with p-value <0.2.

## Results

**Figure 1:**
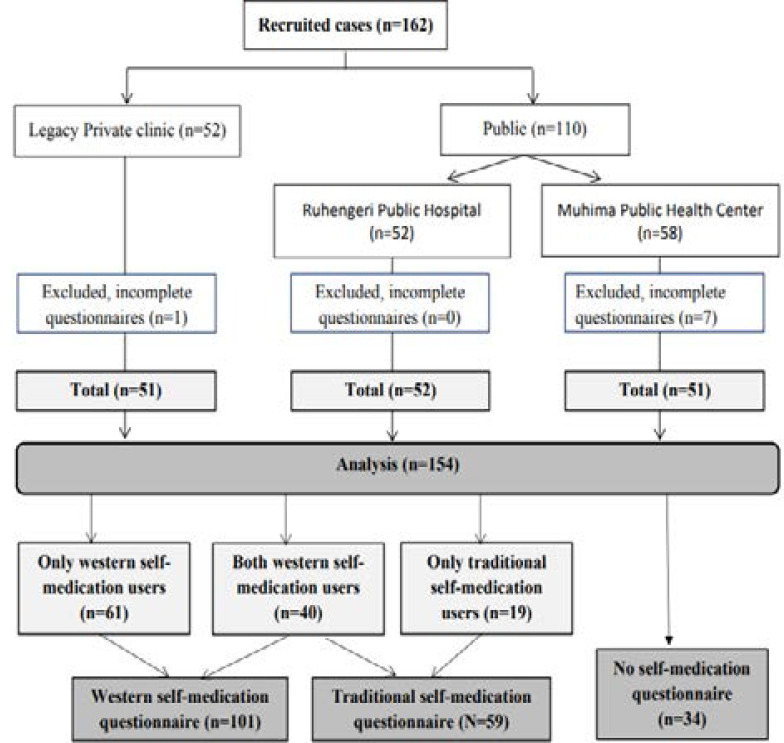
Consort flow diagram (n=number of respondents, N=Number of participants)

**Study period:** Recruitment took place from July to September 2018 with all sites being visited twice, gaining a total of 162 subjects who were enrolled (Figure 1).

**Participation flow and social-demographic characteristics:** Eight participants were excluded from the analysis as they did not complete the questionnaires fully, leaving a total of 154 participants who were included in the final analysis with complete data. Fourteen participants (9%) self-completed the questionnaire with 140 (91%) needing assistance with verbal completion of the questionnaire. The mean age of respondents was 33 years (SD: 6.7). Mothers represented the majority (86.4%) of respondent and most respondents were married (94%) (Table 1).

**Table 1:** Participant demographics

Item	Classification	Frequency	Percentage
**Site of interview**	Private	51	33.1%
	Public	103	66.9%

**Origin**	Kigali	92	59.7%
	Outside Kigali	62	40.3%

**Relationship**	Mother	133	86.4%
	Other	21	13.6%

**Marital status of parent**	Married	145	94.2%
	Single (+ Separated, Widowed)	9	5.8%

**Age of parent**	≤30	60	39%
	>30	94	61%

**Education level**	Advanced	77	50%
	Minimal	77	50%

**Economic status**	High	45	29.2%
	Low and Middle	109	70.8%

**Health insurance**	Private	60	39%
	Mutuelle de sante	89	57.8%
	None	5	3.2%

**Number of children under** **10yrs**	1	48	31.2%
	>1	106	68.8%

Advanced education level = completed secondary school or university; Minimal education level = no education or completed primary school; High economic status = have both employment and own house; Middle economic class = have either employment or own house; Low economic class = have neither employment nor own house;

Self-medication indications and practices: Among 154 participants, 120 caregivers (78%) reported to have used self-medication for their children, Sixty-one of them (51%) used only modern drugs in self-medication, 19 (16%) used only traditional drugs while 40 (33%) used both types. Thirty-four caregivers had never used self-medication (22%) (Figure 1).

Many parents/caregivers decided to self-medicate with modern medications without seeking advice from anyone. Whereas, for traditional self-medication, friends and relatives served as the main sources of advice (Table 2). All traditional self-medication users used local Rwandan herbs. Paracetamol was the most commonly used modern medicine. Traditional medicine was most commonly used when parents/caregivers suspected intestinal worms.

**Table 2:** Self-medication indications and practice

Item	Classification	Modern medicines (N=101)	Traditional medicine (N=59)
Source of advice before deciding to use self-medication	Myself	54 (53.4%)	14 (23.7%)
	Health care provider	32 (31.7%)	0 (0%)
	Friend/relative	17 (16.8)	36 (61.0%)
	Internet/media	5 (5.0%)	5 (8.5%)
	Traditional healer	1 (1.0%)	11 (18.6%)
	Other	2 (1.9%)	-

Symptom for which self-medication is given	Fever	78 (77.2%)	3 (5.1%)
	Cough and runny nose	44 (43.6%)	16 (27.1%)
	Intestinal worms	15 (14.8%)	20 (33.8%)
	Pain	6 (5.9%)	1 (1.7%)
	Difficult breathing	5 (5.0%)	2 (3.4%)
	Diarrhea/vomiting	5 (5.0%)	13 (22.0%)
	Skin conditions	3 (3.0%)	5 (8.5%)
	Others	15 (14.8%)	17 (28.8%)

Source of medications	Pharmacy	94 (93.1%)	-
	Leftover medicine	15 (14.9%)	-
	Health care facility w/o prescription	5 (5.0%)	-
	Shop/store	1 (1.0%)	-
	Relative/friend	0 (0%)	5 (8.5%)
	Traditional healer	-	22 (37.3%)
	Self-collected herbs /plants	-	36 (94.7%)
	Others	-	2 (5.2%)

Type of Health care facility consulted in case of self-medication failure	Private	47 (46.5%)	9 (15.3%)
	Public	38 (37.6%)	23 (39.0%)
	Traditional healer	5 (5.0%)	5 (8.5%)
	None	24 (23.7%)	26 (44.1%)

Drugs used in self-medication with modern medications (n=101)	Paracetamol	73 (72.3%)	-
	Cough Syrup	40 (39.6%)	-
	Ibuprofen	29 (28.7%)	-
	Antibiotics	18 (17.8%)	-
	Drugs for intestinal worms	17 (16.8%)	-
	Skin preparation	4 (4%)	-
	Oral Rehydration Solution (ORS)	3 (3%)	-
	Others	15 (14.8%)	-

Respondents could choose more than two options.

**Reasons for using self-medication:** There was a statistically significant difference in reasons for choosing modern and traditional self-medication among our participants, with participants choosing modern medications having slightly more confidence in self-medication than self-medication providers of traditional medicines (p=0.005) and parents/caregivers who used modern self-medication reporting barriers to consultation more frequently as reason to self-medicate than parents/caregivers who used traditional self-prescription (p=0.028) (Table 3).

**Table 3:** Reasons of using self-medication – Means of Likert questions

	Modern Medicine (n=101)			Traditional medicine (n=59)					p-value
	Disagree	Neutral	Agree	Mean (±SD)	Disagree	Neutral	Agree	Mean (±SD)	
**Confidence in self-prescribing**									
The symptoms of my child are minor and do not require a health facility consultation	29 (28.8%)	0 (0%)	72 (71.2%)	3.56 (±1.08)	23 (40%)	1 (1.7%)	35 (59.3%)	3.31 (±1.14)	p=0.164
I can recognize my child's disease based on his/her symptoms	35 (34.7%)	0 (0%)	66 (65.3%)	3.39 (±1.07)	27 (45.8%)	0 (0%)	32(54.2%)	3.22 (±1.21)	p=0.477
I have used drug previously	24 (23.8%)	1 (1%)	76 (75.2%)	3.64 (±1.04)	24 (40.8%)	0 (0%)	35 (59.3%)	3.31 (±1.22)	p=0.111
I want to give emergency treatment to my child	13 (12.9%)	1 (1%)	87 (86.1%)	3.83 (±0.81)	31 (52.6%)	0 (0%)	28 (47.4%)	3.00 (±1.17)	p<0.001
Non-prescription medicines work well	27 (26.7%)	6 (6%)	68 (67.3%)	3.37 (±1.02)	20 (34%)	3 (5%)	36 (61%)	3.27 (±1.04)	p=0.558
Non-prescription medicines are not dangerous	28 (27.8%)	4 (4%)	69 (68.3%)	3.38 (±1.01)	22 (37.3%)	3 (5%)	34 (57.7%)	3.24 (±1.10)	p=0.441
**Total confidence score**	-	-	-	21.2 (±3.25)	-	-	-	19.3 (±4.38)	p=0.005
**Barriers to** **consultation**									
I don't have health insurance	89 (88.1%)	0 (0%)	12 (11.9%)	2.06 (±1.00)	51 (86.4%)	0 (0%)	8 (13.6%)	2.14 (±0.99)	p=0.484
Consultations fees are high	88 (87.1%)	2 (2%)	11 (10.9%)	2.06 (±0.83)	53 (89.8%)	1 (1.7%)	5 (8.5%)	2.08 (±0.81)	p=0.725
I spent lot of time waiting when I consult a health facility	60 (59.4%)	2 (2%)	39 (38.6%)	2.84 (±1.18)	44 (74.6%)	1 (1.7%)	14 (23.7%)	2.53 (±1.04)	p=0.106
The health facility is far from my home	81 (80.2%)	0 (0%)	20 (19.8%)	2.34 (±0.89)	54 (91.5%)	0 (0%)	5 (8.5%)	2.14 (±0.60)	p=0.250
I don't have time to go to a health facility	71 (70.3%)	1 (1%)	29 (28.7%)	2.53 (±1.11)	53 (89.8%)	0 (0%)	6 (10.2%)	2.03 (±0.76)	p=0.004
Total barriers score	-	-	-	11.8 (±3.18)	-	-	-	10.9 (±2.69)	p=0.028

Likert scale: 1=strongly disagree; 2=disagree; 3=neutral;4=agree; 5=strongly agree. P-value: Mann-Whitney U-test Comparison of means

Non-self-prescribers: Parents/caregivers who choose not to self-medicate reported most frequently that they don't use self-medication because “non-prescription drugs are dangerous”. Access to pharmacy, traditional healer, traditional medication shop or cost, were not found to be reasons to not use self-medication (Table 4).

**Table 4:** Reason for not using self-medication (n=34)

Reasons	Disagree	Neutral	Agree	Mean (±SD)
**Nonprescription drugs are dangerous**	6 (17.7%)	1 (2.9%)	27 (79.4%)	3.82 (±0.96)
**Nonprescription drugs do not work**	9 (26.5%)	3 (8.8%)	22 (64.7%)	3.53 (±1.05)
**My child/children did not get sick**	30 (88.2%)	0 (0%)	4 (11.8%)	2.18 (±0.93)
**I have limited access to pharmacy**	33 (97.1%)	0 (0%)	1 (2.9%)	1.94 (±0.48)
**I can't afford medication in pharmacy**	32 (94.1%)	0 (0%)	2 (5.9%)	1.88 (±0.68)
**I have limited access to traditional** **medication shop/store**	32 (94.1%)	1 (2.9%)	1 (2.9%)	1.82 (±0.62)
**I have limited access to traditional healer**	33 (97.1%)	0 (0%)	1 (2.9%)	1.76 (±0.51)

Likert scale: 1=strongly disagree; 2=disagree; 3= neutral;4=agree; 5=strongly agree

**Social demographic association with use of non-prescription drug:** The association between different demographic characteristics and the use of self-medication was analyzed. In bivariate analysis the factors that were statistically significantly associated with use of non-prescription drugs included being from Kigali and having more than one child less than 10 years of age. In the multivariate analysis of factors that were significant in the bivariate analysis (with cut-off p<0.2), the only factor associated with increased use of self-prescribed drugs was having more than 1 child under the age of 10 years (Table 5).

**Table 5:** Social demographic association with use of nonprescription drug

		Use of medication w/o prescription					

		Yes (n=120)	No (n=34)	OR (CI)	P-value	Adjusted OR (CI)	P-value
**Number of children** **<10yrs**		N (%)	N (%)				

	>1 child	92 (86.8%)	14 (13.2%)	4.6 (2.10–10.48)	p<0.001	4.74 (1.94–11.58)	p=0.001
	One child	28 (58.3%)	20 (41.7%)				

**Origin**	Kigali	78 (84.8%)	14 (15.2%)	2.65 (1.21–5.78)	p=0.013	2.23 (0.91–5.46)	p=0.078
	Outside Kigali	42 (67.7%)	20 (32.2%)				

**Site of interview**	Private	44 (86.3%)	7 (13.7%)	2.23 (0.89–5.55)	p=0.080	1.09 (0.32–3.67)	p=0.886
	Public	76 (73.8%)	27 (26.2%)				

**Economic status**	High	39 (86.6%)	6 (13.3%)	2.24 (0.86–5.87)	p=0.094	1.79 (0.53–6)	p=0.342
	Low/Moderate	81 (74.3)	28 (25.7)				

**Age of parent**	>30	77 (81.9%)	17 (18.1%)	1.79 (0.83–3.86)	p=0.136	0.82 (0.32–2.09)	p=0.679
	<30	43 (71.7%)	17 (28.3%)				

**Education level**	Advanced	61 (79.2%)	16 (20.8%)	1.16 (0.54–2.49)	p=0.699	NA	NA
	Minimal	59 (76.6%)	18 (23.4%)				

**Relationship**	Mother	104 (78.2%)	29 (21.8%)	1.12 (0.37–3.31)	p=0.837	NA	NA
	Other	16 (76.2%)	5 (23.8%)				

**Marital status**	Married	113 (77.9%)	32 (22.1%)	1.00 (0.20–5.09)	p=0.991	NA	NA
	Single	7 (77.8%)	2 (22.2%)				

**Health insurance**	Private	47 (78.3%)	13 (21.7%)	1.04 (0.47–2.27)	p=0.922	NA	NA
	Mutuelle de santé/None	73 (77.7%)	21 (22.3%)				

OR=Odds ratio; AOR=Adjusted Odds Ratio; Multivariate analysis (AOR) undertaken on factors with p-value <0.20

**Association of socio-demographic factors with the use of modern versus. Traditional medication:** The association between socio-demographic factors and the use of modern only vs. traditional only drugs was analyzed. In bivariate analysis the factors associated with use of exclusive modern rather than exclusive traditional drugs in self-medication were: caregivers in private clinic, caregivers from Kigali, advanced education, high social economic class, having private health insurance, and being older than 30 years. e included all factors with a p-value<0.2 in a multivariate analysis and after adjusting for confounders only age above 30 and living in Kigali remained associated with the preference of exclusive modern self-medication compared to exclusive traditional self-medication (Table 6).

**Table 6:** Social demographic association with type of self-medication used(modern vs. traditional)

		Modern only (n=61)	Traditional only (n=19)	OR (95%CI)	p-value	Adjusted OR(CI)	p-value
		N (%)	N (%)				
**Site of** **interview**	Private	33 (97.1%)	1 (2.9%)	21.21 (2.66–169)	p<0.001	0.61 (0.02–18.16)	p=0.778
	Public	28 (60.9%)	18 (39.1%)				

**Education** **level**	Advanced	43 (95.5%)	2 (4.4%)	20.3 (4.24–97.12)	p<0.001	2.38 (0.24–22.92)	p=0.453
	Minimal	18 (51.4%)	17 (48.6%)				

**Economic** **status**	High	30 (96.7%)	1 (3.2%)	17.41 (2.18–138.77)	p=0.001	4.75 (0.32–70.28)	p=0.257
	Low /Moderate	32 (63.2)	18 (36.7)				

**Health** **insurance**	Private	38 (95%)	2 (5%)	14.04 (2.96–66.42)	p<0.001	2.78 (0.20–37.44)	p=0.440
	Mutuelle de santé/none	23 (57.5%)	17 (42.5%)				

**Age of** **parent**	>30	49 (89.1%)	6 (10.9%)	8.84 (2.78–28.08)	p<0.001	5.78 (1.25–26.68)	*p=0.025*
	≤30	12 (48%)	13 (52%)				

**Origin**	Kigali	46 (90.2%)	5 (9.8%)	8.5 (2.65–27.82)	p<0.001	8.2 (1.58–43.12)	*p=0.012*
	Outside Kigali	15 (51.7%)	14 (48.2%)				

**Number of** **children** **<10yrs**	>one	49 (79%)	13 (21%)	1.88 (0.59–5.98)	p=0.281	NA	NA
	One	12 (66.6%)	6 (33.3%)				

**Relationship**	Other	11 (84.6%)	2 (15.4%)	1.87 (0.37–9.29)	p=0.442	NA	NA
	Mother	50 (74.6%)	17 (25.4%)				

**Marital** **status**	Married	59 (76.6%)	18 (23.4%)	1.63(0.14–19.14)	p=0.693	NA	NA
	Single	2 (66.7%)	1 (33.3%)				

OR=Odds ratio; AOR=Adjusted Odds Ratio; Multivariate analysis (AOR) undertaken on factors with p-value <0.20

## Discussion

This study aimed to determine the reported self-medication use in children in private and public health facilities in Rwanda and to determine attitudes and reasons associated with parental decisions to self-medicate their children. Our study showed that 78% of participants have used self-medication for their children. Both modern and traditional drugs were used. This is similar to 77% reported in Pakistan 7 and higher than the prevalence of 32% and 58.82% reported in Madagascar and India 8,11,16. However, higher prevalence of 95.1% and 98.1% were reported in Greece and Australia 17,18. This shows that self-medication in children is a universal practice with some differences between countries.

In our study 51% of parents/caregivers who used self-medication used only modern medicines, 16% used only traditional self-medicines and 33% used both. Our findings show that we had a lower rate of parents/caregivers who used only modern medicines compared to previous studies conducted in Sudan and Saudi Arabia where 84% and 86.7% of parents/caregivers reported using modern medicines over traditional ones 23,24; this difference can be explained by the fact that in our study we separately analyzed participants who used both types of self- medication, and did not attempt to specify whether they preferred modern or traditional drugs.

Self-medication has many potential benefits, for example medications such as paracetamol can be effectively used. However, self-medication can result in ineffective medications being used, wasting parental resources and potentially delaying a child receiving appropriate care. Cough syrups were the second most widely used self-medication. This is of concern, particularly in young children as cough suppressants in this age group are known to be ineffective and to have potentially harmful effects^27^. Drugs for intestinal worms were the third most commonly used in self-medication. Clinical experience of the authors demonstrates that parents/caregivers may be attributing many other symptoms such as decreased appetite, poor weight gain as being caused by intestinal worms and therefore use of drugs for suspected intestinal worms without prior consultation may be inappropriate. Most alarming in our results was the finding that nearly 20% of parents/caregivers were self-medicating their children with antibiotics. As the world aims to combat antibiotic resistance, this form of self-medication should be addressed.

There is a difference between education and economic factors associated with the choice to self-medicate. Studies from Spain, Finland, Italy, and Madagascar^15^,^16^,^28^,^29^, found that families with high income and with secondary and higher education level practice more self-medication than families with low income and low education level. On the other hand in a study done in India, family income and parental education were not significantly influencing self-medication practice, in Germany, mother with lower education level used fewer OTC and no significant difference was seen with regard to income status 11,30. In our study, financial, insurance or geographical access barriers were not reasons to choose self-medication. This finding is not surprising as 58% of our participants had Mutuelle de santé insurance, and access to healthcare in Rwanda is overall good with 79% of the population subscribing to an affordable community health insurance (Mutuelle de Santé) which covers 90% of consultation fees in public health facilities 31.

Furthermore, our study showed that having more than one child less than 10 years of age was associated with the practice of self-medication. No other factor was shown to be associated with parental use of self-medication. Caregiver's age above 30 years of age were found to be associated with the use of modern self-medication compared to traditional ones. In contrast to our findings, being female and younger were associated with the practice of self-medication in Italy and Spain 15,28.

We observed that coming from Kigali city was associated with the preference of modern drugs over traditional ones, which can be explained by the fact that most of our study participants were from Kigali where there is a wider availability of pharmacies dispensing modern medicines than in areas outside Kigali.

## Limitations

Our analysis to identify associated factors did not include the group of parents/caregivers who used both types of self-medication. The internal validity could have been compromised by the high rate of verbal completion of the questionnaire, with participants being potentially prone to acquiescence bias. Recall bias may have also limited caregiver responses. All regional provinces of Rwanda were not represented.

## Conclusion

Self-medication, a worldwide practice, is also common in Rwanda. Parents/caregivers are involved in this practice for their children regardless of their socio-demographic background. Consideration should be given to regulating drugs used in self-medication as well as the education of the population with the goal of minimizing the risks of self- medication and maximizing benefits.

## List of acronyms of terms

CMHS: College of Medicine and Health Sciences
CU: Christian Umuhoza
HC: Health Center
IRB: Institutional Review Board
JU: Joyeuse Ukwishaka
NM: Natalie McCall
NSAID: Non-Steroidal Anti-Inflammatory Drugs
OPD: Out Patient Department
OTC: Over the Counter
PC: Peter Cartledge
PI: Principal Investigator
UTHK: University Teaching Hospital of Kigali
WHO: World Health Organization
